# Soil properties predict below-ground community structure, but not nematode microbiome patterns in semi-arid habitats

**DOI:** 10.1111/mec.17501

**Published:** 2024-08-22

**Authors:** Tiago José Pereira, Alejandro De Santiago, Holly M. Bik

**Affiliations:** 1 Department of Marine Sciences, University of Georgia, Athens, Georgia, USA; 2 Institute of Bioinformatics, University of Georgia, Athens, Georgia, USA

**Keywords:** metabarcoding, microbiome, nematodes, semi-arid habitats, soil biodiversity, soil bulk density

## Abstract

Microbial and microeukaryotic communities are extremely abundant and diverse in soil habitats where they play critical roles in ecosystem functioning and services that are essential to soil health. Soil biodiversity is influenced by above-ground (vegetation) and below-ground factors (soil properties), which together create habitat-specific conditions. However, the compound effects of vegetation and soil properties on soil communities are less studied or often focused on one component of the soil biota. Here, we integrate metabarcoding (16S and 18S rRNA genes) and nematode morphology to assess the effects of habitat and soil properties shaping microbial and microeukaryotic communities as well as nematode-associated microbiomes. We show that both vegetation and soil properties (soil bulk density) were major factors structuring microbial and microeukaryotic communities in semi-arid soil habitats. Despite having lower nutrients and lower pH, denser soils displayed significantly higher alpha diversity than less dense soils across datasets. Nematode-associated microbiomes have lower microbial diversity, strongly differ from soil microbes and are more likely to respond to microscale variations among samples than to vegetation or soil bulk density. Consequently, different nematode lineages and trophic groups are likely to display similar associated microbiomes when sharing the same microhabitat. Different microbiome taxa were enriched within specific nematode lineages (e.g. *Mycobacterium*, *Candidatus Cardinium*) highlighting potentially new species-specific associations that may confer benefits to their soil nematode hosts. Our findings highlight the importance of exploring above- and below-ground effects to assess community structure in terrestrial habitats, and how fine-scale analyses are critical for understanding patterns of host-associated microbiomes.

## 1 | INTRODUCTION

Soils are extremely diverse habitats, harbouring ~59% of the species on Earth (Anthony et al., 2023). Many taxa, including archaea, bacteria, fungi, viruses, protists and other microeukaryotes such as nematodes, are highly diverse and abundant in these systems (Guerra et al., 2021; van den Hoogen et al., 2019). In fact, below-ground biomass equals or potentially exceeds that above-ground, highlighting the importance of small organisms living within the soil (FAO et al., 2020). Furthermore, soil organisms play fundamental roles in maintaining soil health and stability through recycling and remineralization of nutrients, waste decomposition and carbon sequestration (Bahram et al., 2018; Bardgett & van der Putten, 2014).

Soil biodiversity is regulated by both above- and below-ground factors (Islam et al., 2020; Wardle et al., 2004). For instance, plant species and biomass directly influence the availability of carbon and other nutrients in the soils, thus impacting the diversity, distribution and community composition of bacteria and fungi (Alberti et al., 2017; Islam et al., 2020). Plant traits including leaf area and leaf nitrogen content can also regulate the diversity and community composition of bacteria and fungi (Moreau et al., 2015; Thion et al., 2016), while phosphorus content appears to have a narrower influence on bacterial communities and bacterivorous nematodes (Wang et al., 2022). Soil physicochemical parameters including soil texture, organic matter, pH and nutrients are also known to influence the spatial distribution of both microbes and microeukaryotes (Alberti et al., 2017; Islam et al., 2020; Quist et al., 2019). In particular, soil compaction (e.g. soil bulk density) is directly related to soil porosity which regulates air permeability, water infiltration, nutrient flux, root penetration and biological activity and therefore strongly affects soil biota (Beylich et al., 2010; Hartmann et al., 2014; Labouyrie et al., 2023). Given the high biodiversity and complexity of soil habitats, it can be difficult to untangle large-scale versus localized factors impacting microbial community patterns.

Soil community assembly also appears to be influenced by both organismal body size and spatial scale (Chen et al., 2020; Luan et al., 2020; Quist et al., 2017; Wang et al., 2022). A recent global meta-analysis has suggested a general relationship between organismal body size and the influence of stochastic vs. deterministic processes shaping community assembly, where the smallest size classes (bacteria/archaea) are influenced by dispersal-based stochastic processes while community assembly in larger microbial eukaryotes (fungi, protists and nematodes) is primarily influenced by selection-based deterministic processes (Luan et al., 2020). However, the abundance and diversity of soil biota as well as the processes shaping them may vary drastically depending on the spatial scale (Bardgett & van der Putten, 2014; Kuramae et al., 2012), resulting in situations where small-scale habitat heterogeneity (i.e. between centimetres to metres) can have a stronger impact on structuring soil biodiversity compared with broad environmental drivers. As a result, the spatial distribution of soil organisms can be very patchy. For example, Armstrong et al. (2016) showed that for microbial communities in desert habitats, spatial variation has a much stronger effect on species composition than temporal variation, mainly due to the soil heterogeneity. Similarly, Bakonyi et al. (2007) showed that microhabitats have a strong effect on the structure of soil nematode communities, even greater than warming treatment conditions.

Soil microeukaryotes such as nematodes and their associated microbiomes have not been well studied when compared to bacteria/archaeal assemblages and large above-ground plant and animal species (Anthony et al., 2023; de Araujo et al., 2018; Lara et al., 2022). Nematodes are the most abundant terrestrial metazoans reaching thousands of individuals per 100 g of dry soil and filling critical roles in the soil food web (Ettema, 1998; Ferris et al., 2001; van den Hoogen et al., 2019). Nematodes are also species-rich, represent various trophic groups and have been widely used as bioindicators in diverse ecosystems (Bongers & Bongers, 1998; Nielsen et al., 2014; van den Hoogen et al., 2019). Nematode-associated microbiomes (i.e. endo- or ectosymbionts, transient or resident gut taxa, and microbes attached to mucus secretions and cuticle surface) represent another hidden source of soil biodiversity that may contribute significantly to nematode parasitism in animals and plants (Alves et al., 2018; Lo et al., 2024; Topalović & Vestergård, 2021), nematode fitness and tolerance to abiotic conditions (Derycke et al., 2016; Dirksen et al., 2016; Zhang et al., 2021), and overall ecosystem functioning (Zhu et al., 2021). Previous studies have shown that the associated microbiomes of nematodes and other invertebrates are distinct and less diverse than the surrounding environmental microbes (Boscaro et al., 2022; Schuelke et al., 2018; Zheng et al., 2020), and appear to also be driven by host group and host trophic level in soils (McQueen et al., 2022; Zhu et al., 2021). Yet, studies focusing on nematode-associated microbiome patterns have been limited to a few taxa (Dirksen et al., 2016; McQueen et al., 2023; Zheng et al., 2019). Therefore, understanding the additional roles of nematode-associated microbiomes—and how these associations are further shaped by above- and below-ground factors—remains an important avenue of research.

In the present study, we carried out metabarcoding of bacteria/archaea (16 rRNA from soil), microeukaryotes (18S rRNA from soil) and nematode-associated microbiomes (16S rRNA profiles from individual worms) to assess the effects of above-ground (vegetation) and below-ground (soil properties) factors in structuring soil biodiversity in the Shipley-Skinner Reserve in Southern California. By carrying out a replicated sampling strategy across distinct habitats with varying degrees of geographic proximity, we aimed to assess whether overarching community patterns for soil microbes and microeukaryotes would also be reflected in the nematode-associated microbiomes. We hypothesized that (1) soil community assemblages across all taxonomic levels will be mainly structured by below-ground factors and that soil diversity will vary across habitats, (2) nematode-associated microbiomes will be mainly driven by small-scale habitat heterogeneity rather than broader factors such as soil density and vegetation, and (3) nematode phylogeny and feeding group will not influence nematode-associated microbiomes. To the best of our knowledge, this is the first study that attempts to undertake a simultaneous assessment of nematode-associated microbiomes alongside the characterization of soil biodiversity patterns across multiple domains of life, providing novel insights on how above- and below-ground factors shape soil communities with distinct life histories and environmental requirements.

## 2 | MATERIALS AND METHODS

### 2.1 | Study area

**FIGURE 1:**
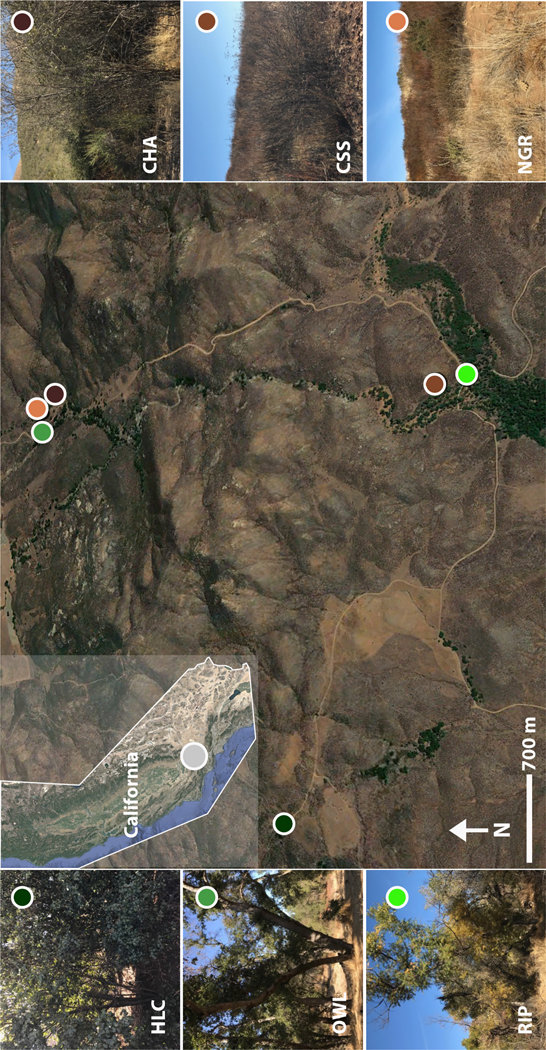
Study area. Samples were collected at the Shipley-Skinner Reserve in Southern CA, US. Brown and green circles indicate soils with higher and lower soil bulk density (BD), respectively. Soil habitats: Chaparral (CHA), coastal scrub sage (CSS), native grass (NGR), holly-leaf cherry (HLC), oak woodland (OWL) and riparian (RIP). Inset: CA map showing the location of Shipley-Skinner Reserve (grey circle).

The Shipley-Skinner Reserve was established in 1992 in Southern California, US (33°39′18″ N, 116°59′49″ W), within the Western Riverside County Multi-Species Habitat Reserve to provide habitat connectivity between the Skinner Reservoir and Diamond Valley Lake (Figure 1). The reserve is characterized by fragmented semiarid plant communities and a Mediterranean climate with short cool winters and long, hot, dry summers. Rain is mostly restricted to the winter months, with a mean annual precipitation of ~25 cm (Lee & Rotenberry, 2015; Shates et al., 2018). Dominant habitats include coastal sage scrub, non-native grasslands and chaparral. Other smaller habitats include holly-leaf cherry, native grasslands, coast live oak woodland, southern willow scrub and live oak, and cottonwood willow riparian forests (Table S1). These habitats, however, are rapidly disappearing due to wildfires and urbanization in Southern California, thus affecting the distribution of both above- and below-ground species (Beyers et al., 1995; Cox & Allen, 2007; Lee & Rotenberry, 2015).

### 2.2 | Sampling and sample processing

Samples were collected on 18 December 2017, across three shrub/grassland habitats (chaparral, coastal sage scrub and native grasslands) and three wooded habitats (holly-leaf cherry, oak woodland and riparian; Figure 1; Table S1). Three composite replicates (i.e. made of three cores collected within <1 m of each other) were taken from each habitat with a metal corer (4.7 cm diameter × 14 cm depth) to characterize soil communities. Replicates were collected at ~3 m of distance from each other to account for within-habitat heterogeneity. Three additional soil samples (about 200 g) were collected from each habitat for soil analyses: soil texture (Sand%, Silt% and Clay%), soil bulk density (BD), pH, organic matter (OM), organic carbon (OC) and nutrients (N: nitrogen, P: phosphorus and K: potassium; Table S1). All environmental analyses were performed at the UC Davis Analytical Laboratory, except for BD, which was performed at the Agricultural and Environmental Services Laboratories, University of Georgia (see Table S1 for additional information regarding the methods used for physicochemical analysis of soils). Samples were kept cool during sampling and immediately transported to the laboratory at UC Riverside once sampling was completed. In the laboratory, soil samples collected for biodiversity assessment were properly mixed and split into similar amounts (~100 g) for molecular and morphological work. Samples used for molecular analyses were stored at −80°C until processed.

### 2.3 | Nematode-associated microbiomes

Nematodes were extracted from ~100 g soil using the Baermann Funnel technique (Viglierchio & Schmitt, 1983). Specimens were recollected from funnels after 24 hrs and 48 hrs, and then transferred to 50 mL falcon tubes for long-term preservation at −80°C. From each sample, 30 specimens were randomly picked under a dissecting microscope (Olympus SZX16, Olympus Corporation, Tokyo, Japan), washed three times in molecular-grade water following the methods of Derycke et al. (2016), morphologically identified (family or genus level) and imaged on temporary slide mounts under a compound light microscope (Nikon Eclipse E600; Nikon Corporation) prior to DNA extraction and PCR procedures (Pereira et al., 2020). A total of 540 nematodes were used to examine patterns in the nematode-associated microbiomes and to provide complementary data regarding soil nematode communities (e.g. to confirm the taxonomic assignments of abundant metabarcoding Amplicon Sequencing Variants [ASVs] using Sanger-generated DNA barcodes of taxonomically identified nematode specimens; Table S2). The remaining material from the Baermann Funnels was concentrated in 1.5 mL PCR tubes and stored at −80°C for future work.

### 2.4 | Metabarcoding of soil and nematode-associated microbiome samples

We extracted genomic DNA from soil samples (0.25 g of soil) using the ZymoBIOMICS^™^ DNA Miniprep Kit (catalogue nos.: D4300, Zymo Research Corp, Irvine, CA) following the manufacturer’s directions. Three technical DNA extraction replicates were generated from each soil sample, for a total of 54 DNA extractions (6 habitats × 3 samples per habitat × 3 technical replicates per sample). Blank samples (kit controls containing water instead of soil samples, Table S3) were also included during the DNA extraction process to account for potential kit contamination. Lysates were stored at −80°C until PCR was carried out. We amplified the 16S rRNA gene from bacteria/archaea (515F/806R primers targeting the V4 region; Caporaso et al., 2012) and 18S rRNA gene from microbial eukaryotes (F04/R22 primer targeting the V1–V2 regions; Creer et al., 2010). Both rRNA genes were amplified using the reagents and PCR conditions in the Earth Microbiome Project (EMP) protocols (Caporaso et al., 2012). Dual-index primer constructs were designed by modifying the EMP Illumina amplicon protocol (Thompson et al., 2017). Details on 16S and 18S rRNA primer constructs are given in Schuelke et al. (2018), and oligo sequences are available on FigShare (https://doi.org/10.6084/m9.figshare.5701090). Nematode-associated microbiomes were extracted from single specimens following the methods of Schuelke et al. (2018), using a simple worm lysis buffer (WLB) containing proteinase K. Briefly, nematodes were recovered from temporary slides, placed on a new sterile glass slide containing 5 μL of WLB and transferred into a 200-μl PCR tube containing an additional 20 μL of WLB. Taxonomy blank samples (i.e. those containing only WLB, Table S3) were also included as a checkpoint for potential sources of contamination in the laboratory. Nematodes were incubated in a Thermomixer heated shaker block (Eppendorf, Hamburg, Germany) at 65°C and 750 rpm for 2 h, followed by a 5-min incubation at 100°C (Schuelke et al., 2018). Lysates were used immediately or stored at −20°C. Nematode-associated microbiomes were amplified using the 16S rRNA metabarcoding primers described above, where microbes from single-worm DNA extractions served as the starting template. All metabarcoding PCRs were set up in a dedicated laminar-flow hood that underwent daily sterilization with bleach and UV light. Metabarcoding PCRs had a final volume of 25 μL and contained 1 μL of DNA template, 0.5 μL of each primer (10 μM), 10 μL of Platinum Hot Start PCR Master Mix (2×) (Thermo Fisher) and 13 μL of molecular-grade water. Both positive (ZymoBIOMICS Microbial Community Standard, Zymo Research, Irvine, CA) and negative controls (molecular-grade water) were included in all PCRs.

The following PCR profile was used for the amplification of both 18S and 16S rRNA genes: 94°C for 3 min; 94°C for 45 s, 50°C for 60 s and 72°C for 90 s for 35 cycles; and 72°C for 10 min. PCR amplification success was evaluated via gel electrophoresis (agar 1%) to confirm gel bands of the expected fragment size. PCR purification was subsequently carried out using a magnetic bead purification protocol with Agencourt AMPure XP beads (Beckman Coulter, CA, USA) and following the manufacturer’s protocol. Sample DNA concentrations were measured using a Qubit^®^ 3.0 Fluorometer and a Qubit^®^ dsDNA HS (High Sensitivity) Assay Kit (Thermo Fisher Scientific), and normalization values were calculated to ensure that equivalent DNA concentrations were pooled across all samples.

Metabarcoding libraries were separately pooled and subjected to a final magnetic bead cleanup step on the final pool, followed by size selection on a BluePippin (Sage Science, Beverly, MA) to remove any remaining primer dimer and isolate target PCR amplicons within the range of 300–700 bp. A Bioanalyzer trace was run on the size-selected pool as a quality control measure, and 18S and 16S rRNA libraries were sequenced in two separate runs on the Illumina MiSeq Platform (2 × 300-bp paired-end runs) at the UC Davis Genomics Core Facility. PCR negative and positive controls as well as blank samples were also submitted for sequencing alongside soil and single-worm nematode samples (Schuelke et al., 2018). All wet laboratory protocols and downstream bioinformatics scripts used in this study have been deposited on GitHub (https://github.com/BikLab/shipley-skinner).

### 2.5 | Generating nematode reference sequences via Sanger sequencing

We used Sanger sequencing to generate 18S rRNA barcodes (~1600 bp) from a subset of 132 nematodes selected for the characterization of the nematode-associated microbiomes (Table S2). These sequences were further used to improve the molecular reference databases used for taxonomic assignment of eDNA metabarcoding reads. The 18S rRNA gene was amplified using three overlapping PCR primer sets (G18S4/R26, 22F/13R and 24F1/18P; Bik et al., 2010; Blaxter et al., 1998). PCR reactions were 25 μL total volume, containing 3 μL of nematode DNA template, 1 μL of each primer (10 μM), 12 μL of Q5^®^ Hot Start High-Fidelity 2x Master Mix (New England Biolabs) and 8 μL of molecular-grade water (HyClone HyPure Water, GE Healthcare Life Sciences). The following PCR profile was used for amplification of all three fragments: 98°C for 30 s; 98°C for 10 s, 55.4°C for 30 s and 72°C for 30 s for 35 cycles; and 72°C for 2 min. Amplification success was evaluated via electrophoresis on a 1% agarose gel stained with SYBR^®^ Green. Successful PCRs were purified with Agencourt AMPure XP beads (Beckman Coulter) using an in-house magnetic bead cleanup protocol, and fragments sequenced in both directions using ABI-PRISM^®^ Dye-DeoxyTerminator Big DyeTM v3.1 (Applied Biosystems) on an automatic Gene Analyzer^®^ ABI 3100 sequencer (Applied Biosystems) at the Institute for Integrative Genome Biology at UC Riverside. Newly obtained 18S rRNA sequences were concatenated and quality-checked using CodonCode Aligner v. 4.2.7 (CodonCode Corporation, LI-COR, Inc.) following the methods in Pereira et al. (2020). DNA barcode sequences generated in this study have been deposited in GenBank (accession nos.: PP099577–PP099708).

### 2.6 | Illumina data processing and generation of ASVs

Raw Illumina data were demultiplexed using a custom script for handling dual-index barcode combinations. Demultiplexed 16S and 18S rRNA datasets were analysed in QIIME2 version 2023.9 (Bolyen et al., 2019) where primer sequences were trimmed using the *cutadapt* plugin (Martin, 2011). Denoising was based on optimal parameters (forward and reverse reads truncated at 237 and 253 bp for 16S rRNA and 245 and 287 bp for 18S rRNA, with a median PHRED score of ≥30). Subsequently, ASVs were generated using DADA2 with default parameters (Callahan et al., 2016), including default chimera checking parameters (de novo chimera identification).

Taxonomy assignments of 16S and 18S ASVs were obtained with the *BLAST+* consensus taxonomy classifier (minimum confidence value of 0.8; Camacho et al., 2009). For the 16S rRNA dataset, the SILVA 138 SSURef NR99 release was used to assign taxonomy (Quast et al., 2013). For the 18S rRNA dataset, we used a custom reference database that included sequences from the 138 SSURef NR99 release (Quast et al., 2013), short nematode 18S rRNA sequences from Macheriotou et al. (2019), full-length nematode 18S rRNA sequences from Pereira et al. (2020) and full-length 18S rRNA sequences generated from soil nematodes via Sanger sequencing as part of this study.

### 2.7 | Bioinformatics and statistical analyses

Samples with low read count (i.e. after demultiplexing and *DADA2*) were not included in our downstream analyses (Table S3). Based on a trade-off between sequencing depth and sample size, final datasets were composed of 50 samples (read count ≥ 4000 reads) for the 16S rRNA soil; 45 samples (read count ≥ 1000 reads) for the 18S rRNA soil; 520 samples for the 16S rRNA nematode-associated microbiome (read count ≥ 100 reads). To assess the levels of contamination across different datasets, we used the R package *decontam* v1.20 (Davis et al., 2018) with the prevalence method and a contaminant classification threshold of 0.5. Sequences determined to be contaminants were removed from the datasets before further analyses were carried out (Table S3).

Contaminant-filtered ASV tables were used to assess soil microbial and microeukaryotic community patterns associated with soil bulk density (i.e. lower and higher soil BD) and habitat (vegetation). Alpha-diversity estimates including observed diversity, Shannon diversity *H*′ (Log_2_), Inverted Simpson (*D*) diversity and Pielou’s Evenness (*J*′) were calculated using *phyloseq* v1.44 (McMurdie & Holmes, 2013) and compared among habitats and soil BD. Data normality was assessed using Shapiro–Wilk’s method, and Kruskal–Wallis (K-W) tests were used to assess differences among factors with the package *FSA* v0.8.24 in *R* v4.2.2 (R Core Team, 2021). The Mann–Whitney *U*-test with adjustments for *p*-value (BH method; Benjamini & Hochberg, 1995) was used for pairwise comparisons (Zar, 2010). Alpha diversity was also explored with barplots based on the relative abundance of dominant taxa at different taxonomic levels.

To visualize the similarity of soil microbial (16S rRNA) and microeukaryotic (18S rRNA) communities associated with the different soil habitats, a similarity matrix based on Bray–Curtis similarity index and ASV-transformed abundances (i.e. standardized by total and square root transformed) was constructed. Ordination was completed using non-metric multidimensional scaling (nMDS) and goodness-of-fit given by the stress value (Clarke, 1993). Permutational analysis of variance (PERMANOVA) was used to test for significance among soil habitats (Anderson et al., 2008). Differential abundance analyses were performed independently for each dataset using the package *ALDEx2* v1.32 (Fernandes et al., 2013, 2014). ASV counts were centred-log ratio (CLR) transformed for a compositionally coherent inference and estimates. Significant differences (*p* < .05) among soil habitats were assessed through K–W tests at each taxonomic rank. False discovery rates (FDRs) were estimated using the BH procedure (Benjamini & Hochberg, 1995).

For the 16S rRNA nematode-associated microbiome dataset, we also assessed the effects of broader factors (i.e. habitat and soil BD) in structuring nematode-associated microbiomes. Furthermore, we tested for the effects of small spatial scale variation (i.e. among samples), nematode phylogeny and nematode feeding ecology using smaller datasets (i.e. specific soil habitats and nematode groups). Thus, soil nematode groups well sampled in this study (e.g. aphelenchids, cephalobids, dorylaimids, plectids, rhabditids and tylenchids; Table S2) were analysed separately. Differences in the nematode-associated microbiomes among samples and nematode groups were illustrated using the biplots produced after canonical analysis of the principal coordinates (CAP) based on the same distance/similarity matrices used for PERMANOVA. Alpha diversity among nematode taxonomic and feeding groups was assessed as described above. Patterns related to nematode phylogeny and feeding groups were explored via nMDS and by using the package *ggtree* v3.8.2 in *R* (Yu et al., 2018).

Principal components analysis (PCA) was performed on a Euclidean distance matrix based on the environmental variables after the removal of highly correlated ones (i.e. *R* = >0.95) to characterize soil habitats. For this study, all visualizations were produced with *ggplot2* v.3.4.4 in *R* (Wickham, 2016).

## 3 | RESULTS

### 3.1 | Shipley-Skinner Reserve: Environment and soil communities

**FIGURE 2:**
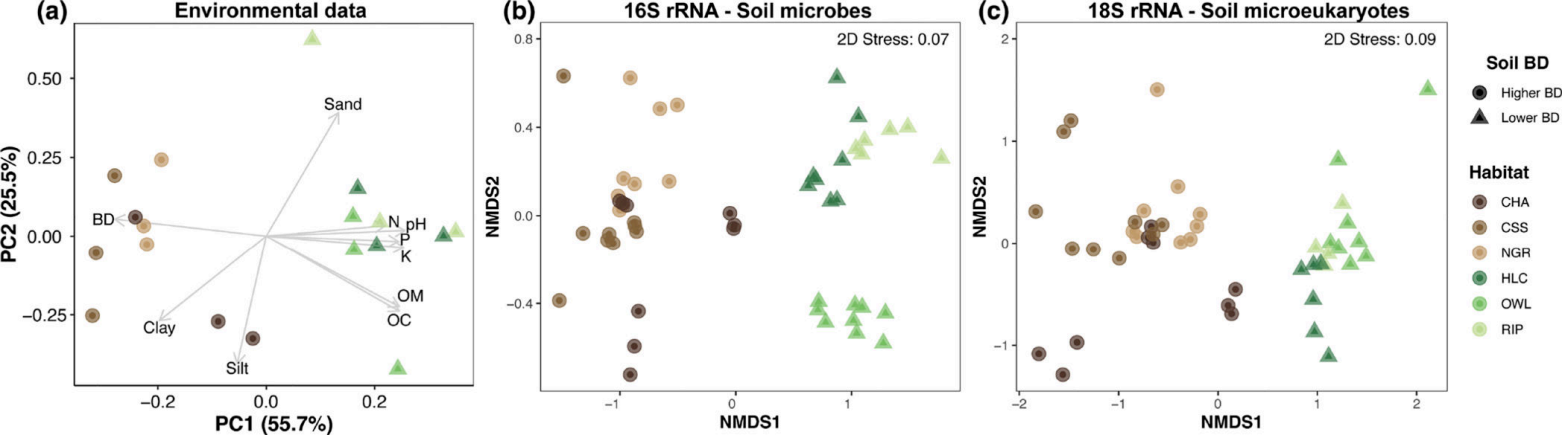
Ordinations based on (a) environmental, (b) 16 rRNA and (c) 18S rRNA datasets. Principal components analysis (PCA) is based on the Euclidean distance from normalized environmental variables. nMDSs are based on the Bray–Curtis similarity constructed from the relative abundance (square root transformed) of ASVs. Soil habitats: chaparral (CHA), coastal scrub sage (CSS), native grass (NGR), holly-leaf cherry (HLC), oak woodland (OWL) and riparian (RIP). Shades of brown and green indicate habitats having higher (circle) and lower (triangle) soil bulk density (BD), respectively.

Environmental analyses clearly separated soil samples into two distinct groupings reflecting differences in soil properties. Soils in the shrub/grassland habitats (chaparral, coastal sage scrub and native grass) displayed higher values of BD, Silt% and Clay%, while wooded habitats (holly-leaf cherry, oak woodland and riparian) exhibited higher pH, nutrient levels and OM (Figure 2a). Accordingly, PC1 and PC2 together explained 81.2% of the total variation among samples. PERMANOVA analysis detected significant differences between soil BD (Pseudo-*F* = 12.5, P(MC) = 0.0007), but not among habitats (Table S4). Furthermore, significant differences between soil BD were detected for all environmental variables, except Silt% (KW analysis; Figure S1; Table S1).

Alpha diversity associated with microbial and microeukaryotic soil communities varied significantly among habitats and between soil BD levels (Table 1). For soil microbes, significant differences among habitats (after FDR with BH method) were observed for Shannon, Simpson and evenness with Oakwood land displaying the lowest values. For Simpson diversity, holly-leaf cherry also displayed significantly lower values than chaparral, coastal sage scrub and riparian. For soil microeukaryotes, significant differences among habitats were only observed for Simpson diversity (native grass showing the highest mean value) and evenness (native grass greater than oak woodland, Table 1). Overall, soils with higher BD displayed higher soil biodiversity, and this was consistent for both bacterial/archaeal (16S rRNA) and microeukaryote (18S rRNA) datasets (Table 1).

Soil communities across all taxonomic groups were strongly structured by soil BD and vegetation (Figure 2b,c). PERMANOVA analysis detected significant differences among all habitats, except between native grass and chaparral in the 16S bacterial/archaeal dataset (Table S4). Overlap among habitats was more evident in denser soils, especially for the 18S microeukaryote dataset (Figure 2c), thus resulting in lower within habitat similarity when compared to less dense soils (16S rRNA: 37% vs. 44%; 18S rRNA: 31% vs. 41%). Variation among samples (i.e. small scale) within soil habitats was also observed for both microbial and microeukaryotic communities (Table S5).

### 3.2 | Major soil microbial and microeukaryotic taxa

Soil microbial communities were dominated by (i) *Acidobacteriota* (7%–23%) with *Vicinamibacteriales* (4%–20%) more abundant in less dense soils; (ii) *Actinobacteriota* (17%–38%) with *Frankiales* (1%–12%) more abundant in denser soils and *Solirubrobacterales* (4%–12%) commonly found across all habitats; (iii) *Firmicutes* (4%–21%) with *Bacillales* (2%–19%), especially abundant at oak woodland; (iv) and *Proteobacteria* (22%–37%) with *Alphaproteobacteria* (11%–22%) and *Gammaproteobacteria* (6%–22%) more important in soils having higher and lower BD, respectively (Figure S2). Differentially abundant taxa displaying the highest abundances and enriched at less dense soils included *Bacillus*, *Planococcus* and *Vicinamibacterales*, especially at oak woodland; *Solirubrobacter* and *Vicinamibacteraceae*, especially at riparian; *Nitrososphaeraceae*, especially at holly-leaf cherry. Enriched taxa at denser soils included *Actinobacteriota* strain 67–14, *Blastococcus* and *Sphingomonas*, especially at coastal sage scrub. Other less abundant taxa, but highly significant with respect to their abundance across soil habitats included *Achromobacter*, *Acidibacter* and *Streptosporangium*, all enriched in less dense soils, and *Afipia* and *Angustibacter* enriched in denser soils (Table S6).

Fungi (41%–91%) including *Dothideomycetes* (11%–55%), *Eurotiomycetes* (9%–37%) and *Sordariomycetes* (5%–35%)-dominated microeukaryotic 18S rRNA reads. *Agaricomycetes* (1%–37%) was especially abundant at oak woodland, whereas *Pezizomycotina* was restricted to denser soils (Figure S2). Metazoans were more abundant in less dense soils and mostly represented by nematodes, tardigrades and collembolans. Other metazoan groups (e.g. Arachnida) were less abundant or habitat-specific (e.g. Insecta). Archaeplastida including Chlorophyceae and Streptophyta were mostly restricted to denser soils, whereas eukaryotic SAR lineages, especially Cercozoa, were recovered in lower abundance across all habitats (Figure S2). Examples of differentially abundant eukaryotic taxa included *Verticillium*, *Penicillium*, *Tylocephalus* and Qudsianematidae, enriched at less dense soils; *Sclerotinia*, *Knufia*, *Rhogostoma* and Trebouxiophyceae, enriched in denser soils (Figure S2; Table S7).

### 3.3 | Nematode soil diversity and nematode-associated microbiomes

A total of 41 nematode genera, representing 20 nematode families, were morphologically identified. Cephalobids (bacterivore, 28.3%), aphelenchids (fungivores, 20.7%) and tylenchids (fungivores/plant parasites, 16.9%) were the most common taxa (Figure S3). Dorylaimids (predators, fungivores and omnivores; 13.2%), rhabditids (bacterivore 12.2%) and plectids (bacterivore, 6.7%) were less abundant, while others were considered rare (~2%). *Acrobeles* (15.2%), *Acrobeloides* (8%), *Aphelenchus* (10.6%), *Aphelenchoides* (8.7%) and *Aporcelaimellus* (9.6%) were the most common nematode genera accounting for ~52% of all identified specimens (Table S2). Bacterivore nematodes including cephalobids (e.g. *Acrobeles*), panagrolaimids (e.g. *Panagrolaimus*), plectids and rhabditids were mostly abundant in less dense soils (46%–74%), whereas fungivores (e.g. *Aphelenchus* and *Aphelenchoides*) were more common at denser soils (13%–64%). Herbivores were habitat-specific with Tylenchidae found at native grass (up to 60%) and Dolichodoridae at oak woodland (up 21%), whereas the predator *Aporcelaimellus* displayed similar abundances across soil habitats (Figure S3).

**FIGURE 3:**
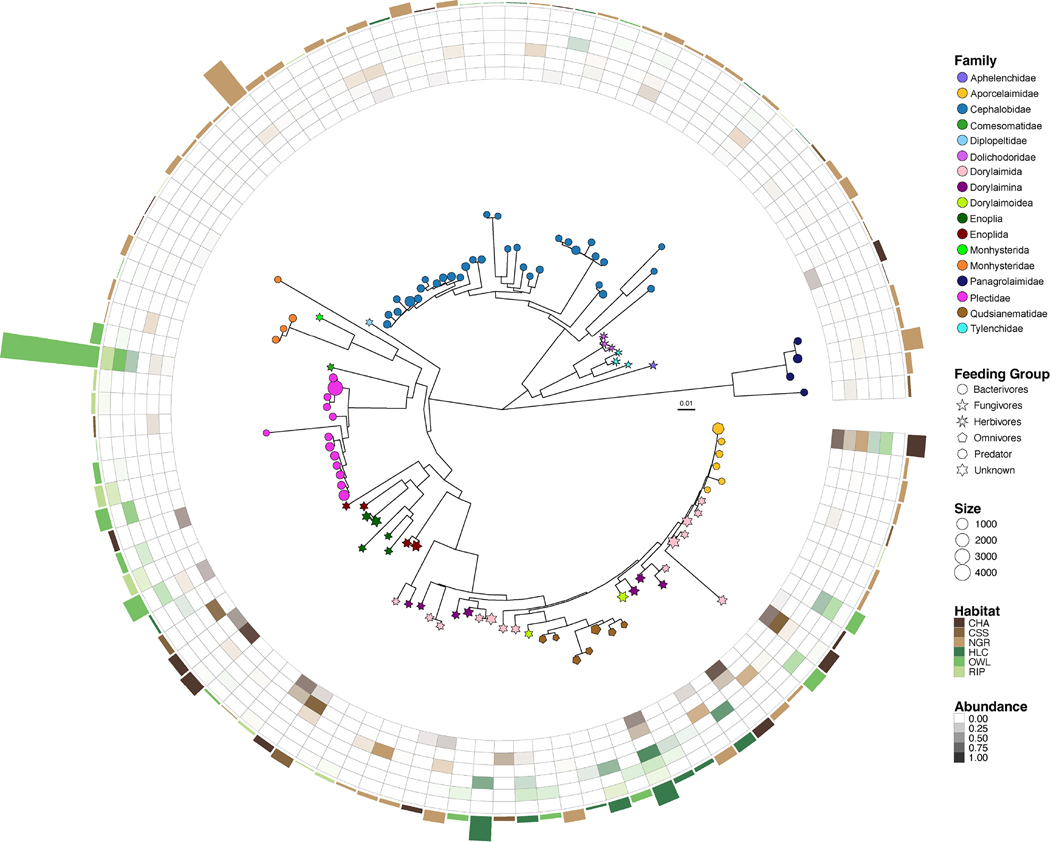
18S rRNA nematode phylogenetic tree based on 109 ASVs. Habitat prevalence (i.e. habitat with the total highest abundance) for each nematode ASV is given in the outer circle (barplot), whereas the relative abundance of ASVs across habitats and soil bulk density (Higher BD: brown shades; Lower BD: green shades) is given in the inner circle. Nematode taxonomy and trophic groups are given by colour code and symbols, respectively. Shape size indicates the total abundance of nematode ASVs. Soil habitats: chaparral (CHA), coastal scrub sage (CSS), native grass (NGR), holly-leaf cherry (HLC), oak woodland (OWL) and riparian (RIP).

A total of 109 ASVs matching Nematoda was retrieved from the 18S microeukaryote dataset with dorylaimids (e.g. Dorylaimida and Qudsianematidae) and plectids (e.g. *Plectus* and *Tylocephalus*) displaying the highest abundances (Figure 3). Overall, nematode ASVs were habitat-specific leading to variation within trophic groups: for example, bacterivore plectids were more abundant in habitats with lower BD whereas bacterivore cephalobids and panagrolaimids were more abundant in denser soils (Figure 3). Only three ASVs matching *Aporcelaimellus*, Qudsianematidae and *Tylocephalus* were broadly distributed across soil habitats. The 18S rRNA metabarcoding approach recovered 11 nematode genera including some of the abundant taxa in the morphological dataset (e.g. *Acrobeles*, *Acrobeloides*, *Tylocephalus* and *Aporcelaimellus*). Finer taxonomy assignment of ASVs matching dorylaimids and enoplids (rare in the morphological dataset) was precluded, thus impacting the nematode feeding group proportions in the molecular dataset (i.e. ‘unknowns’; Figure S3).

**FIGURE 4:**
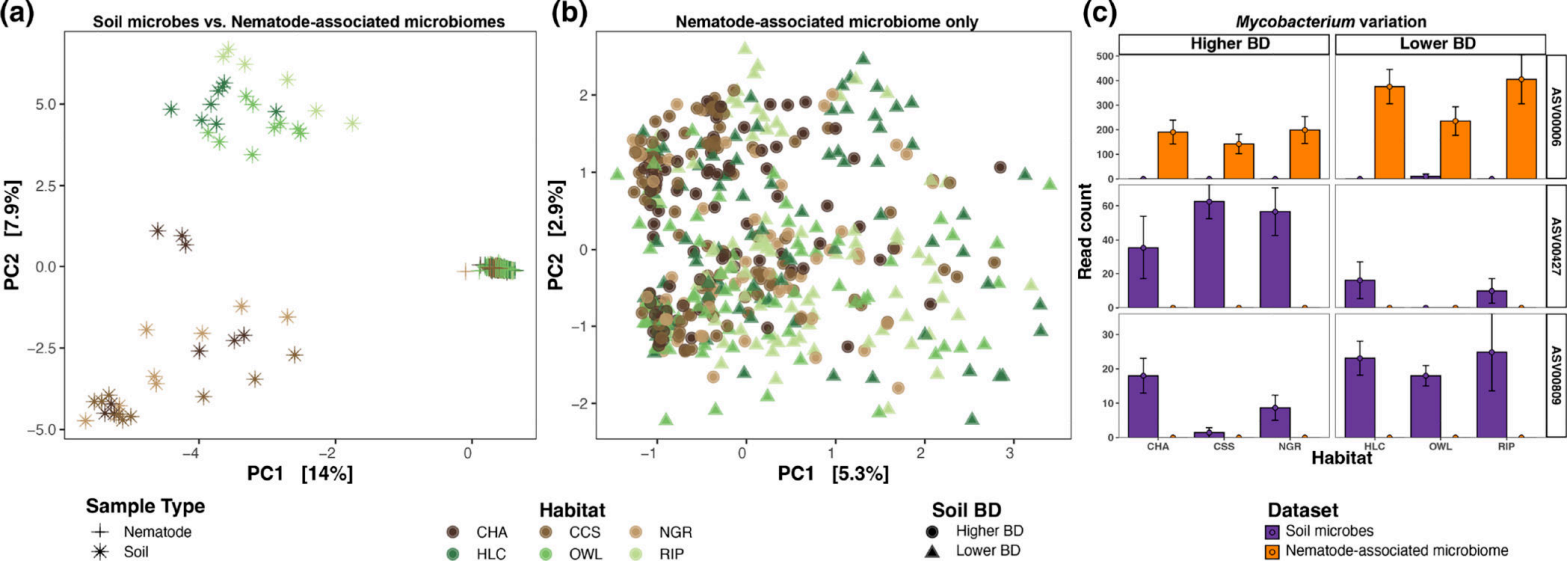
Ordinations based on (a) the entire 16S rRNA dataset (soil microbes vs. nematode-associated microbiomes) and (b) 16S rRNA nematode-associated microbiomes only. Principal components analysis (PCAs) are based on the Euclidean distance from the CLR transformation of AVSs abundance. (c) Variation in the abundance of *Mycobacterium* ASVs found in the soil (purple) and nematode-associated microbiomes (orange) across soil habitats. Soil habitats: Chaparral (CHA), coastal scrub sage (CSS), native grass (NGR), holly-leaf cherry (HLC), oak woodland (OWL) and riparian (RIP). Shades of brown and green indicate habitats having higher (circle) and lower (triangle) soil bulk density (BD), respectively.

Nematode-associated microbiomes grouped distinctly from bulk soil microbial assemblages; however, no patterns related to soil BD and vegetation were recovered (Figure 4a,b). In contrast with soil microbial communities, nematode-associated microbiomes displayed lower abundances of *Acidobacteria* (<5% across all samples) and *Actinobacteriota* (5%–18%) but higher abundances of *Firmicutes* (10%–34%) and *Proteobacteria* (25%–64%, especially *Gammaproteobacteria*; Figure S4). *Bacteroidota* (2%–48%) was also important, especially in tylenchids found in native grass which displayed a high percentage of *Cytophagales*. Nematode-associated microbiomes across different taxonomic groups were often dominated by the same bacterial genera (Table S8). Particularly, the genus *Mycobacterium* (ASV0006) was the most abundant (mean reads per group 171–376) and frequent (30%–60% of specimens in a group) microbe in all nematode taxonomic groups except rhabditids where *Lacunisphaera* (ASV00011) was the most abundant (mean reads 52) and frequent (30%). The microbial *Candidatus Cardinium* (ASV00001) was especially abundant (mean reads 4989) and frequent (30%) in tylenchids, but poorly recovered or absent in other nematode taxonomic groups (Table S8). Although *Mycobacterium* was represented by multiple ASVs, *Mycobacterium*_ASV0006 was almost entirely restricted to the nematode-associated microbiome (frequency: 2% vs. 51%), whereas other abundant and frequent *Mycobacterium* ASVs (e.g. ASV00427 and ASV00809) were only recovered in soil samples (Figure 4c). Similar to *Mycobacterium*_ASV0006, *Lacunisphaera*_ASV00011 and *Candidatus Cardinium*_ASV00001 were almost entirely restricted to the nematode-associated microbiomes (Table S8).

The 16S bacterial/archaeal alpha diversity from soils was much higher than that found in the nematode-associated microbiomes. In multiple cases, the nematode-associated microbiome was only represented by one or two ASVs. Differently from the soil dataset, nematode-associate microbiome alpha diversity was higher in less dense soils, especially from holly-leaf cherry nematodes (Table 1). Nematode-associated microbiome alpha diversity was lower in herbivore nematodes (i.e. Tylenchidae and Dolichodoridae) than all other feeding groups (data not shown).

**FIGURE 5:**
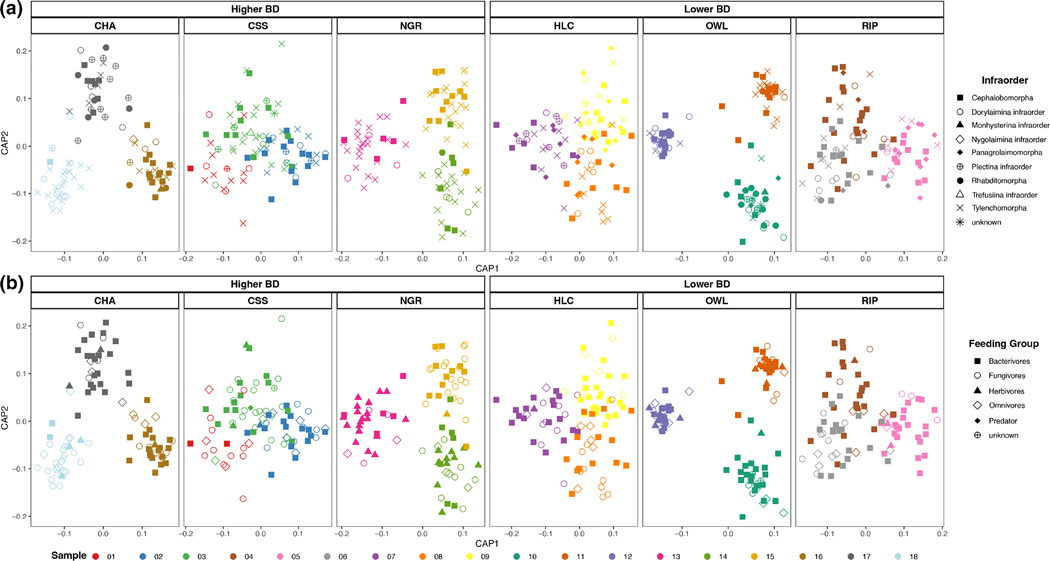
Nematode-associated microbiome structure according to samples within habitat and soil bulk density (higher and lower BD) groups represents (a) nematode infraorder taxonomic rank and (b) feeding groups. CAP is based on the Euclidean distance from the centred-log ratio (CLR) transformation of AVSs abundance. Soil habitats: Chaparral (CHA), coastal scrub sage (CSS), native grass (NGR), holly-leaf cherry (HLC), oak woodland (OWL) and riparian (RIP).

**FIGURE 6:**
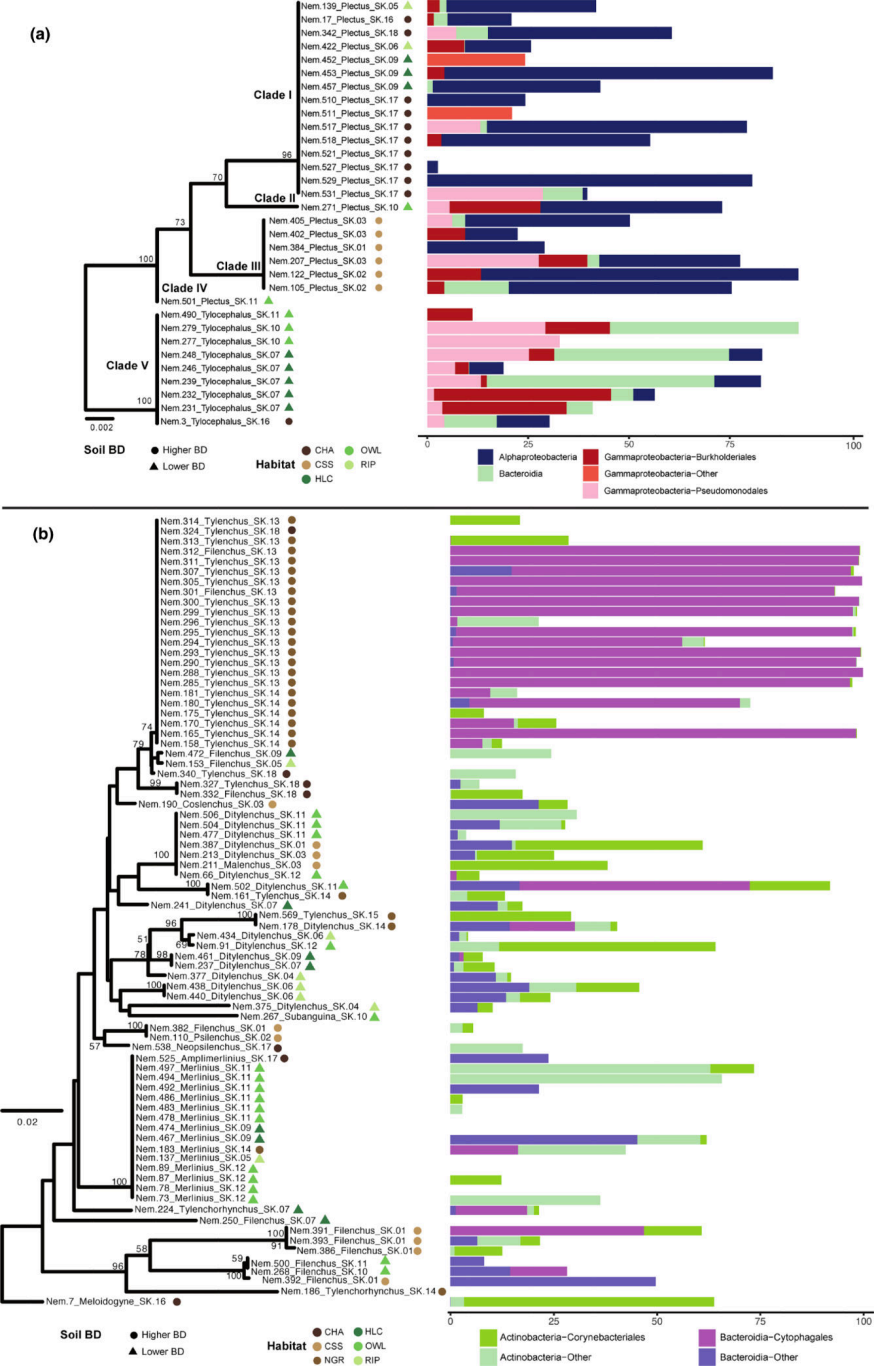
Specific nematode-microbial associations in (a) plectid and (b) tylenchid nematodes. Differences among nematode genera within each group are tested using differential abundance analysis. Phylogenetic trees were built using neighbour-joining (NJ) and p-distance methods with 100 bootstraps (BS ≥ 50% are shown) from representative ASV sequences matching the nematode ID. Plectid tree is rooted on the branch leading to *Tylocephalus*, whereas *Meloidogyne* was used as the root for the tylenchid tree. Taxonomic assignments of nematode-associated microbiomes are colour-coded. Soil habitats: Chaparral (CHA), coastal scrub sage (CSS), native grass (NGR), holly-leaf cherry (HLC), oak woodland (OWL) and riparian (RIP). Soil bulk density (BD) groups: Higher and Lower BD.

Data partitioning by soil habitat showed that nematode-associated microbiomes grouped according to their sampling origin, that is, a ‘sample effect’ (Figure 5). Thus, distantly related nematodes (e.g. different family or genera) as well as different nematode trophic groups displayed a more similar microbiome when found at the same sample. Conversely, when the same nematode taxon and/or trophic group came from different samples, their microbiomes differed drastically (i.e. not structured by high taxonomic rank or feeding group; Figure 5). Based on the nematode-associated microbiomes, significant differences across samples were found in all habitats, except at coastal sage scrub (Table S9). Data partitioning and differential abundance analysis by nematode taxonomic group allowed us to detect variation in the nematode-associated microbiome of plectids, with *Alphaproteobacteria* more abundant in *Plectus* and *Bacteroidia* and *Gammaproteobacteria* (i.e. *Pseudomonadales* and *Burkholderiales*) more abundant in *Tylocephalus* (Figure 6). Similarly, *Acrobeles* and *Acrobeloides* displayed differential abundances for *Alphaproteobacteria* and *Bacilli* whereas *Tylenchus* displayed greater abundances of *Cytophagales* when compared to other tylenchids (Figure 6). Variation among nematode clades (i.e. within the same nematode genus) was observed for *Acrobeles* and *Panagrolaimus* (Table S10).

## 4 | DISCUSSION

### 4.1 | Soil properties and vegetation determine soil community patterns in semi-arid habitats

The most striking pattern recovered in this study was the stark separation of soil communities based on soil properties. Shrub/grass habitats (chaparral, coastal sage scrub and native grass) displayed significantly higher BD (denser soils) and Clay% and significantly lower OM, while tree-dominated habitats (holly-leaf cherry, oak woodland and riparian) were characterized by relatively ‘less dense’ soils exhibiting significantly lower BD and significantly higher Sand% and nutrients. Notably, the grouping of habitats into ‘higher’ and ‘lower’ soil BD was consistent regardless of the geographic distance between samples (Figure 1), indicating that drastic changes in soil biodiversity and community structure can occur locally across very short distances. The above-ground vegetation and below-ground soil properties were also consistent predictors of soil community assemblages across domains of life and body size (i.e. bacteria/archaea and microeukaryotes; Figure 2); however, these environmental factors did not appear to play a role in structuring nematode-associated microbiomes.

Soil compaction is considered a major threat to soil biodiversity, but it has been mostly studied in the context of deforestation, agriculture and urbanization (Beylich et al., 2010; Carlesso et al., 2019; Hartmann et al., 2014; Tibbett et al., 2020). Beylich et al. (2010) assessed the effects of soil compaction on soil organisms and biological processes and concluded that, although highly variable, the effects were only negative when soil BD was >1.7 g cm^−3^. Hartmann et al. (2014) showed that soil compaction due to logging drastically reduced bacteria and fungi abundance, but increased alpha diversity. In an urban environment, Devigne et al. (2016) showed a trend of higher collembolan abundance and alpha diversity at low soil compaction, further supported by significant differences in community structure associated with different levels of soil compaction. In this study, shrubs/grass habitats had significantly higher soil BD (1.07 vs. 1.29 g cm^−3^), but still lower than the harmful threshold proposed by Beylich et al. (2010), thus suggesting that small changes in soil BD may be enough to structure soil communities but unlikely to lower overall soil alpha diversity. Conditions created by soil compaction (e.g. low oxygen availability) can also favour the increased abundance of more adapted taxa (Hartmann et al., 2014). Furthermore, effects of soil compaction may also be group-dependent and potentially more severe in larger soil organisms (Nawaz et al., 2013), which supports the higher diversity of soil nematodes found in less dense soils in our study.

Less dense soils with higher nutrients and OM exhibited consistently lower soil biodiversity across multiple measures (Table 1). Lower microbial diversity and abundance have been associated with higher levels of N (and other nutrients) as it can potentially favour nitrifier communities (Eberwein et al., 2020; Wang et al., 2018). Soil pH, moisture and temperature can also influence soil communities, potentially obscuring the effects of nutrients on soil biota (Adair et al., 2019; Banerjee et al., 2016; Zeng et al., 2016). Soil pH was significantly lower in denser soils (6.34 vs. 7.13), but still characterized as ‘slightly acid’ or ‘neutral’ and therefore close to optimal pH conditions to maintain soil diversity. Different soil organismal groups may thrive in distinct soil pH due to taxon-specific physiological needs. For bacteria, Bahram et al. (2018) showed that both species and functional diversity increase with pH. Although most nematode feeding groups display higher abundances at higher pH, fungivores are more abundant at lower pH soils, potentially due to high fungal abundance (Biswal, 2022; Cesarz et al., 2013). Globally, van den Hoogen et al. (2019) showed that nematode abundance is negatively correlated with soil pH, which suggests that different nematode community metrics may be differently affected by pH.

Vegetation is also an important factor structuring soil communities. For instance, Crowther et al. (2014) found higher species richness of bacteria and fungi in grassland than forest soils, but lower biomass and attributed to the lower abundances of *Acidobacteria* and *Basidiomycota* in the former habitat. In our study, shrub/grass habitats also displayed higher overall soil biodiversity and lower abundances of *Acidobacteria* and *Basidiomycota*, thus suggesting that the high dominance of these two groups can decrease soil diversity. In the Arctic tundra, Chu et al. (2011) showed that although vegetation was the main factor structuring soil communities, its influence varied across soil organismal groups, with stronger effects on bacteria than archaea and fungi. For protists and other microeukaryotes, de Araujo et al. (2018) found significant differences along a vegetation gradient in the Brazilian Cerrado biome and suggested greater microbiome complexity towards tree-dominated vegetation. In agreement with previous studies, our findings also support the prominent role of vegetation in structuring soil microbes and microeukaryotes in semi-arid ecosystems (Figure 2).

Overall, the responses of soil biota to the effects of soil BD and other factors may be group- (e.g. bacteria, fungi and nematodes), metric- (e.g. abundance, alpha- and beta-diversity) and intensity- (e.g. none, low, moderate and strong compaction) dependent. In this Californian semi-arid ecosystem, excessive droughts and wildfires may also increase soil BD, likely impacting soil biodiversity. Studies that further investigate the relationships between vegetation cover, nutrient availability and soil compaction, especially in natural systems, are crucial to improve our understanding of how increased anthropogenic activities may impact microbial and microeukaryote biodiversity and to support restoration projects aiming to recover soil health.

### 4.2 | Environmental filtering explains patterns of nematode taxa among samples and habitats

Both 18S rRNA metabarcoding and morphology (not shown) supported the clustering of soil nematodes based on soil BD and habitats, thus suggesting a strong role of environmental filtering in structuring nematode communities in semi-arid habitats. Environmental filtering, through environmental selection, is a major force structuring soil communities of larger body-size organisms such as nematodes (Luan et al., 2020; Wang et al., 2022; Zinger et al., 2019). At the Shipley-Skinner Reserve, soil nematodes were clearly structured by the abiotic conditions imposed by both above- and below-ground factors. Certain nematode genera, including multiple lineages (i.e. ASVs), were either habitat- or soil-BD-specific (Figure 3). This ‘sample-restricted’ distribution suggests that specific nematode lineages, potentially including cryptic species, are subject to stronger environmental filtering. For instance, plectids were mostly abundant at less dense soils; one putative species, *Tylocephalus*_ASV0342, was found to be abundant in all three lower BD soil habitats, but almost entirely absent from denser soil habitats (Figure 3).

Higher occurrence of plectids have been positively correlated with OM and Sand% (Quist et al., 2019), higher in the less dense soils sampled here. Bacterivores are also associated with high N, potentially due to increased bacterial biomass (Biswal, 2022). This may explain the higher abundance of plectids in riparian and oak woodland habitats as they displayed the highest values of N. In contrast, ASVs assigned to cephalobids, mostly *Acrobeles* and *Acrobeloides*, were more abundant in more dense soils. Bouwman and Arts (2000) showed that soil compaction in grassland agricultural systems had negative effects on nematode species composition and feeding groups, except on cephalobids which may occupy a position in both the rhizosphere and in the soil pores. These patterns show that variation within nematode trophic groups may be due to the differential response of nematode lineages to abiotic factors.

Only two nematode ASVs, matching *Aporcelaimellus* and Qudsianematidae, were broadly distributed across more and less dense soils thus demonstrating a cosmopolitan behaviour (Figure 3). These nematodes are classified as ‘persisters’ and are known to withstand anaerobic conditions and dehydration for long periods (Bongers, 1990), which may help to explain their occurrence in the higher BD soils sampled here. The distribution of *Aporcelaimellus* in soil habitats has also been associated with more suitable temperature and moisture conditions (Bakonyi et al., 2007), which are improved by larger and denser vegetation as seen at tree-dominated soils.

### 4.3 | Nematode-associated microbiomes do not mirror host biodiversity patterns

Nematode-associated microbiomes did not display any patterns related to soil BD, vegetation, feeding group or phylogeny, but strongly differed from the surrounding soil microbes by displaying lower alpha diversity (Table 1; Figure 4), in agreement with previous studies (Boscaro et al., 2022; Schuelke et al., 2018; Zheng et al., 2020). Invertebrate host-associated microbiomes often exhibit lower alpha diversity than their surrounding environmental microbial communities (Boscaro et al., 2022; Tibbs-Cortes et al., 2022; Zheng et al., 2020; Zhu et al., 2021), suggesting some degree of environmental filtering for microbial taxa that become host-associated. Lower alpha diversity in nematode-associated microbiomes may be related to unfavourable conditions in the nematode host (e.g. pH, nutrients and oxygen), which can impose greater selective pressures on microbes acting as an environmental filter (Taylor et al., 2022; Taylor & Vega, 2021; Zhu et al., 2021). In our dataset, herbivorous nematodes displayed even lower alpha diversity in their associated microbiomes compared with other feeding groups, while omnivores and predators, despite lower sample sizes, had higher alpha diversity in agreement with Zhu et al. (2021) who found positive correlations between alpha diversity and trophic levels of soil invertebrates.

Inter-individual variability may also obscure patterns in host-associated microbiomes and introduce an element of stochasticity in terms of which ASVs are recovered via metabarcoding. In this study, the number of ASVs detected across nematodes varied substantially (1–138), with ~50% nematode specimens having = < 10 ASVs. The high dominance of a few ASVs may explain not only the lower diversity in nematode-associated microbiomes but also explain the consistent lack of patterns associated with nematode species (or higher ranks), feeding groups and habitats (e.g. Boscaro et al., 2022; Schuelke et al., 2018; Vafeiadou et al., 2022; Zheng et al., 2020). Inter-individual variability has also been shown to impact nematode-associated microbiomes in controlled laboratory experiments, thus highlighting the importance of sample size when studying microeukaryote-associated microbiomes (Lo et al., 2024; Taylor et al., 2022; Taylor & Vega, 2021). Nevertheless, some microbiome patterns do appear to be emerging: the apparent presence of broad invertebrate-associated bacterial clades including *Rhodobacteraceae* and *Flavobacteriaceae*, and clearer symbiont signals at the genus- and species-level (Boscaro et al., 2022); microbiome patterns indicative of niche partitioning within cryptic species complexes with important changes in the abundances of *Sphingomonadaceae* and *Moraxellaceae*, thus contributing to environmental filtering of the host invertebrates (Derycke et al., 2016). Interesting, these four microbial families reported in marine invertebrate hosts were also important in our study as part of the nematode-associated microbiomes (Table S8).

### 4.4 | Fine-scale analysis reveals spatial patterns and distinct microbiome taxa among closely related nematodes

When the nematode-associated microbiome data was subset and analysed solely per habitat, host-associated microbial assemblages grouped strongly by sample, regardless of host nematode taxa and/or host feeding ecology (Figure 5). Strong sample effects, potentially reflecting microhabitat conditions, were also reported by Zhu et al. (2021) when assessing soil invertebrate microbiomes more broadly. Our findings suggest that even distantly related nematode taxa are likely to share similar microbiomes if they coexist sympatrically, similar to recent findings in marine microscopic invertebrates (Boscaro et al., 2022). Unlike the mammalian gut microbiome with strong phylosymbiosis signal and co-evolution of host/microbiome taxa (Mallott & Amato, 2021), there is currently little to no evidence for phylosymbiosis in terrestrial or marine invertebrates (at least based on analyses at higher taxonomic levels; Boscaro et al., 2022). Our data suggest the existence of an environmental ‘fingerprint’ reflected in host microbiome profiles (potentially reflective of local prey items, or transient microbes ingested into the gut during feeding), alongside parallel species-specific microbiome signals potentially indicative of symbiont or host-associated taxa (discussed further below).

The exact factors influencing microeukaryote-associated microbiomes remains unclear. Our findings indicate that broader factors such as soil BD and vegetation do not obviously shape nematode-associated microbiomes, thus suggesting that other ‘unknown’ factors play a more important role (McQueen et al., 2022; Zhu et al., 2021). Bacterial community assembly in invertebrate hosts may be governed more strongly by functional genes (selecting for metabolic role or products) than species (as defined by 16S rRNA), as has been demonstrated in other biological systems (Boon et al., 2014; Burke et al., 2011; Kost et al., 2023). In the model nematode *Caenorhabditis elegans*, Zimmermann et al. (2020) identified key bacterial traits and necessary metabolic networks that influenced the ability of bacteria to colonize their host. More targeted microbiome studies focusing on gene composition and function (rather than solely on species identity derived via 16S metabarcoding), are needed to determine whether microeukaryote-associated microbiomes are a result of general environmental processes, or whether they represent specialized symbiont taxa that have potentially co-evolved with their hosts. Our nematode microbiome data suggests a mixture of both—stochastic environmental ‘noise’ recovered alongside hidden species-specific microbiome signals.

Four bacterial taxa including *Mycobacterium*, *Rhizobacter*, *Enhydrobacter* and *Candidatus Cardinium* were found to be differentially abundant across major nematode groups (Figure 6; Table S10). *Cardinium* is an obligate endosymbiont broadly found in animals, including small invertebrates, and has been reported in plant-parasitic nematode genera such as *Pratylenchus* and *Heterodera* (Brown et al., 2018; Denver et al., 2016; Santos-Garcia et al., 2014; Yushin et al., 2022). Here, the abundance of *Candidatus Cardinium* was enriched in plant-parasitic nematodes, especially in the genus *Tylenchus* (Figure 6; Table S10). The high prevalence of *Cardinium* in plant-parasitic nematodes suggests that this bacterial group may confer a benefit to its host with an important role in lipid metabolism and biosynthetic capability (Brown et al., 2018; Guo et al., 2022; Yushin et al., 2022). Panagrolaimids displayed significantly higher abundance of *Rhizobacter* when compared to other nematode groups (Table S10). *Rhizobacter* is considered a plant pathogenic bacterium and has been previously isolated from soil, plant roots and freshwater sediments (Jin et al., 2016). As far as we know, *Rhizobacter* has not been yet reported as a potential host-associated microbe of nematodes or other soil invertebrates and it may represent a new nematode-bacterial association with currently unknown functions that will require further analyses to elucidate the potential roles (Geisen, 2021). Other *Comamonadaceae* (e.g. *Acidovorax*, *Comamonas* and *Leptothrix*) were recovered from our nematode-associated microbiome samples, and these have been previously reported as host-associated microbiomes of nematodes (McQueen et al., 2022; Zhang et al., 2017; Zheng et al., 2020), arthropods (Bahrndorff et al., 2018; Kroetsch et al., 2020), ostracods (Schön et al., 2023) and earthworms (Davidson et al., 2013). *Comamonadaceae* has also been reported as a common food source of nematodes and tardigrades in polar habitats (McQueen et al., 2022) and is associated with improved fitness of *C. elegans* (Zhang et al., 2017).

*Mycobacterium* was the most abundant and frequent microbe recovered across nematode-associated microbiome samples, distinguishing nematode-associated ASVs from those found in bulk soil (Figure 4). *Mycobacterium* may include beneficial species that can efficiently stimulate NPK uptake by plants in nutrient-deficient soils, increase soil water infiltration through wax-degrading capabilities, produce phytohormones (auxins), protect against heavy metal exposure or pathogens such as *M. celatum* commonly found in coniferous forest of Europe (Dell’Amico et al., 2008; Islam et al., 2020; Labouyrie et al., 2023; Philippot et al., 2023; Tsavkelova et al., 2005). Bulk soil microbial assemblages at the Shipley-Skinner Reserve displayed low abundances of *Mycobacterium*, particularly so at denser soils. *Mycobacterium* is known to exhibit aerobic lifestyles and thus can be drastically reduced by soil compaction (Longepierre et al., 2021). Still, the fact that *Mycobacterium*_ASV0006 was largely found across nematode microbiome samples in different soil habitats suggests that specific strains within this bacterial genus may be important (and potentially beneficial) associates of soil nematodes.

Using fine-scale analysis of our nematode-associated microbiome dataset, we were also able to detect phylogenetic patterns in the host-associated microbiomes of specific nematode genera (e.g. *Plectus* and *Tylocephalus*; *Acrobeles*, and *Acrobeloides*) as well as among nematode clades within the same genus (e.g. *Acrobeles* and *Panagrolaimus*), thus supporting the existence of distinct species-specific microbiome assemblages in nematodes as previously observed in marine nematodes (Derycke et al., 2016; Vafeiadou et al., 2022). The complexity of host-associated microbiome datasets (and low signal-to-noise ratio) may obscure important signals when carrying out high-level alpha- and beta-diversity analyses that are typically employed in metabarcoding studies. However, our increased sample size combined with the use of phylogenetic frameworks to conduct iterative genus- and ASV-level analyses, suggests that many novel invertebrate-associated microbiome associations can still be discovered using rRNA marker gene datasets.

## 5 | CONCLUSION

In this study, we find that above-ground and below-ground factors are important in shaping the community structure of both bacteria/archaea and microeukaryotes, but these strong patterns do not propagate to the nematode-associated microbiomes. Notably, tree-dominated and less dense soils were less diverse with respect to soil microbial and microeukaryotic communities than grass-dominated and denser soils. Community structure and differential abundance analyses of the 16S bacterial/archaeal and 18S microeukaryote assemblages further supported the distinction between these two major soil habitats. Nematode morphology and 18S rRNA metabarcoding suggest a strong role of environmental filtering in structuring soil nematode communities. Nematode-associated microbiomes were less diverse and distinct from the surrounding soil microbes and were likely shaped by micro-scale soil variation signalling a ‘sample effect’, but we were able to recover a number of species-specific microbiome signals suggestive of symbiont taxa or beneficial microbiome associates. Our findings provide critical insight into how spatial patterns of microbial assemblages may impact nematode-associated microbiomes in semiarid soil habitats, thus contributing towards expanding the current knowledge of host-associated microbiomes in microeukaryotes.

## Supplementary Material

Supplemental material 1

Supplemental material 2

## Figures and Tables

**TABLE 1:** Summary (mean values) of alpha-diversity metrics and number of reads for habitats and soils according to each dataset (16S rRNA: archaea/bacteria; 18S rRNA: microeukaryotes; 16S rRNA: nematode-associated microbiomes).

		Habitats^a^						Kruskal–Walli			Soil BD^b^		Kruskal-Wallis		
					
Dataset	Metrics	CHA	CSS	NGR	HLC	OWL	RIP	*X* ^2^	df	*p*	Higher	Lower	*X* ^2^	df	*p*
16S rRNA: Archaea/Bacteria (Soil)	Number of Reads	18,233	20,637	17,343	16,980	14,533	18,195	6.21	5	.286	18,737	16,339	2.97	1	.085
	Number of ASVs	608.4	668.4	590.1	569.4	460.7	663.83	11.56	5	.041	622.3	551.5	4.42	1	**.036**
	Shannon (*H′*)	5.9	6.0	5.8	5.8	5.4	5.93	20.59	5	**.001**	5.89	5.65	7.69	1	**.006**
	Simpson (*D*)	232.2	254.0	214.6	184.9	90.1	238.94	28.29	5	**<.0001**	233.62	161.89	12.34	1	**.0004**
	Evenness (*J′*)	0.920	0.924	0.920	0.912	0.881	0.921	25.83	5	**.0001**	0.921	0.902	11.80	1	**.0006**
18S rRNA: Eukaryotes (Soil)	Number of Reads	29,637	22,080	24,923	19,057	19,577	17,753	9.50	5	.091	25,547	18,998	6.98	1	**.008**
	Number of ASVs	326.0	230.6	322.8	286.7	301.8	222.8	4.89	5	.429	293.1	279.2	0.11	1	.737
	Shannon (*H′*)	4.6	4.1	4.6	4.5	4.1	3.8	12.20	5	.032	4.4	4.2	2.56	1	.110
	Simpson (*D*)	44.3	30.4	52.3	43.5	19.6	15.4	19.26	5	**.002**	42.3	26.7	5.81	1	**.016**
	Evenness (*J′*)	0.798	0.795	0.820	0.795	0.733	0.741	12.87	5	**.025**	0.8	0.755	7.47	1	**.006**
16S rRNA (Nematode-associated microbiome)	Number of Reads	5643	3784	8989	8832	8035	7276	35.85	5	**<.0001**	6170	8044	5.81	1	**.020**
	Number of ASVs	10.81	9.93	11.36	17.79	15.22	13.51	41.11	5	**<.0001**	10.7	15.5	22	1	**<.0001**
	Shannon (*H*′)	1.63	1.49	1.49	2.03	1.73	1.81	37.13	5	**<.0001**	1.5	1.9	22.8	1	**<.0001**
	Simpson (*D*)	4.94	4.36	4.41	6.82	5.83	5.40	28.88	5	**<.0001**	4.6	6.0	17.6	1	**<.0001**
	Evenness (*J*′)	0.75	0.75	0.65	0.78	0.74	0.76	11.67	5	**.04**	0.71	0.76	4.73	1	**.030**

*Note*: Kruskal–Wallis analysis was used to test for significant differences (*p* < .05) among habitats and between soil bulk density (BD) groups.

a Habitats: chaparral (CHA), coastal scrub sage (CSS), native grass (NGR), holly-leaf cherry (HLC), oak woodland (OWL) and riparian (RIP).

b Soil BD: higher soil bulk density (higher), lower soil bulk density (lower).

Significant differences (*p* < .05) after FDR adjustment (BH method) are highlighted in bold.

## Data Availability

Raw Illumina metabarcoding data (16S and 18S rRNA) have been deposited in the NCBI Sequence Read Archive (BioProject PRJNA1064049). The 18S rRNA gene sequences generated via Sanger sequencing and their morphological IDs have been deposited on GenBank (accession nos.: PP099577–PP099708). The *QIIME2* mapping files, *QIIME2* and *R* outputs, and all scripts used for processing and analysing the data presented in this study are available via GitHub (https://github.com/BikLab/shipley-skinner). Metabarcoding primer constructs for 16S and 18S rRNA genes have been made available on FigShare (https://doi.org/10.6084/m9.figshare.5701090).
