# Supplementary material for: Soil properties predict below-ground community structure, but not nematode microbiome patterns in semi-arid habitats
Source: Mol Ecol. Author manuscript; Available in PMC 2025 Sep 1. (PMC11614143; doi:10.1111/mec.17501)
Supplement: Supplemental material 2 [file NIHMS2031369-supplement-Supplemental_material_2.docx]

**Figure S1.** Mean (± SE) values of environmental variables among habitats (**a-d**) and soil bulk density (BD) groups (Higher and Lower BD) (**e-g**) sampled at Shipley Skinner Reserve. Soil habitats: chaparral (CHA), coastal scrub sage (CSS), native grass (NGR), holly-leaf cherry (HLC), oak woodland (OWL), and riparian (RIP). KW statistical tests and p-value for comparisons between soil BD groups are provided in Table S1.

**Figure S2.** Soil microbial and microeukaryotic community composition across soil habitats. (**a-c**) Phylum, class, and order level classification for bacteria/archaea, respectively. (**d-f**) Major microeukaryotic groups, metazoans, and fungi, respectively. Low abundance taxa were grouped into the “Others” category. Relative abundance of taxa contributing to ≥5% is displayed on the barplots. Soil habitats: chaparral (CHA), coastal scrub sage (CSS), native grass (NGR), holly-leaf cherry (HLC), oak woodland (OWL), and riparian (RIP). Soil bulk density (BD) groups: Higher and Lower BD.

**Figure S3**. Nematode families, genera, and feeding group classifications recovered from morphological/Sanger sequencing (**a-c**) and 18S rRNA soil metabarcoding datasets (**d-f**), respectively. Soil habitats: chaparral (CHA), coastal scrub sage (CSS), native grass (NGR), holly-leaf cherry (HLC), oak woodland (OWL), and riparian (RIP). Soil bulk density (BD) groups: Higher and Lower BD.

**Figure S4.** Most abundant bacterial (**a**) phyla and (**b**) classes retrieved from soil and nematode-associated microbiome samples. Soil habitats: chaparral (CHA), coastal scrub sage (CSS), native grass (NGR), holly-leaf cherry (HLC), oak woodland (OWL), and riparian (RIP). Soil bulk density (BD) groups: Higher and Lower BD.
